# Toward Quantitative Neurosurgical Guidance With High-Resolution Microscopy of 5-Aminolevulinic Acid-Induced Protoporphyrin IX

**DOI:** 10.3389/fonc.2019.00592

**Published:** 2019-07-03

**Authors:** Linpeng Wei, Yoko Fujita, Nader Sanai, Jonathan T. C. Liu

**Affiliations:** ^1^Department of Mechanical Engineering, University of Washington, Seattle, WA, United States; ^2^Department of Neurological Surgery, Barrow Neurological Institute, Phoenix, AZ, United States; ^3^Department of Pathology, University of Washington School of Medicine, Seattle, WA, United States

**Keywords:** fluorescence-guided surgery, handheld microscopy, 5-ALA, PpIX, gliomas, quantitative imaging

## Abstract

Low-power fluorescence microscopy of 5-ALA-induced PpIX has emerged as a valuable intraoperative imaging technology for improving the resection of malignant gliomas. However, current fluorescence imaging tools are not highly sensitive nor quantitative, which limits their effectiveness for optimizing operative decisions near the surgical margins of gliomas, in particular non-enhancing low-grade gliomas. Intraoperative high-resolution optical-sectioning microscopy can potentially serve as a valuable complement to low-power fluorescence microscopy by providing reproducible quantification of tumor parameters at the infiltrative margins of diffuse gliomas. In this forward-looking perspective article, we provide a brief discussion of recent technical advancements, pilot clinical studies, and our vision of the future adoption of handheld optical-sectioning microscopy at the final stages of glioma surgeries to enhance the extent of resection. We list a number of challenges for clinical acceptance, as well as potential strategies to overcome such obstacles for the surgical implementation of these *in vivo* microscopy techniques.

## Introduction

Gliomas are the most common primary malignant brain tumors, with ~20,000 new cases each year in the United States (1). As the standard-of-care for all grades of gliomas, patients generally receive surgery as a first-line treatment. The goal of this “debulking” surgery is to maximize the extent of resection (EOR) while avoiding neurological damage. Mounting evidence suggests that more-extensive EOR is associated with increased overall survival and progression-free survival for both low- and high-grade gliomas patients (2–11). Unfortunately, optimal EOR is not achieved in many patients due to the lack of effective technologies to delineate tumor margins intraoperatively, with reported rates of gross-total resection (GTR) for high-grade gliomas (HGGs) ranging from 33 to 76% (12–19) and for low-grade gliomas (LGGs) ranging from 14 to 46% (7–9, 20, 21).

In recent years, numerous reports have detailed the benefits of using 5-aminolevulinic acid (5-ALA) for guiding HGG resections (22–38). In brief, 5-ALA is a non-fluorescent prodrug that is orally administered to patients several hours prior to surgery. 5-ALA is then intra-cellularly metabolized to form a fluorescent byproduct, protoporphyrin IX (PpIX), a heme-synthesis pathway substrate that accumulates preferentially in glioma cells due to metabolic dysregulation (39–43). In most cases, after 5-ALA administration, the bulk of a HGG tumor emits visible red fluorescence (~630 nm) when excited with blue (~405 nm) illumination, as viewed by the unassisted eye or with a wide-field fluorescence surgical microscope. A landmark phase III trial in Europe by Stummer et al. demonstrated that the use of this technique resulted in higher rates of GTR (65 vs. 36%) and 6-month progression-free survival (41 vs. 21.1%) compared to control patients (37). Intraoperative 5-ALA-induced fluorescence has since emerged as a valuable adjunct for HGG surgeries (22, 35–38), and has recently been approved by the US Food and Drug Administration (FDA) for neurosurgical guidance in 2017 (44).

In spite of its clear benefits, 5-ALA-based fluorescence-guided surgery (FGS) suffers from a number of shortcomings. First, it remains ineffective for guiding the resections of most LGGs and at the infiltrative margins of all diffuse gliomas (low- and high-grade) due to the fact that PpIX accumulation in these tissues is typically below the detection limit of conventional low-power wide-field surgical microscopes. Second, the visible fluorescence generated by PpIX is interpreted subjectively (45) and is difficult to quantify. This is because the visualized fluorescence is greatly affected by light-tissue interactions such as absorption and scattering, as well as detection parameters such as the angle and working distance of the microscope (46, 47). In light of these concerns, spectroscopy-based methods have been developed to provide a more accurate and reproducible measurement of the absolute concentration of PpIX expression in tissue, in which mathematical models are used to correct for the aforementioned confounding effects (22, 48–51). These quantitative detection methods should enable more-objective decision-making during tumor resection since the degree of PpIX accumulation has been shown to correlate with proliferative index, mitochondrial content, and other clinicopathologic metrics (22, 23, 40, 52, 53). However, while probe-based spectroscopy can provide improved sensitivity to detect weak PpIX fluorescence (i.e., in LGGs or at the tumor margins of all gliomas) in comparison to conventional wide-field surgical microscopy (54), spectroscopic approaches are typically limited to sampling localized points of tissue at low spatial resolution rather than generating an image over an extended field of view (FOV).

As a high-resolution, high-contrast imaging technique, handheld confocal microscopy has been explored as an alternative solution to guide the resection of both LGGs and HGGs. In 2011, a pilot study by Sanai et al. first demonstrated the feasibility of using a handheld *in vivo* confocal microscope to detect PpIX expression in LGGs (55), in which conventional wide-field surgical microscopy lacked the sensitivity to detect the PpIX fluorescence. Although the raw intensity of PpIX fluorescence, as previously mentioned, is subjective and cannot be reliably quantified, the spatial distribution (e.g., size, density, localization, etc.) of the signal can potentially serve as a reproducible tumor biomarker (56). Most recently, high-speed handheld confocal microscopy with video-mosaicking capabilities have also been developed and are continuing to be refined for 5-ALA-based FGS (57). In this perspective article, we outline a vision for a clinical workflow in which quantitative high-resolution microscopy is implemented to achieve optimal EOR for the ultimate benefit of patients suffering from LGGs and HGGs. We also describe key challenges to overcome and potential strategies to facilitate the clinical acceptance of these new technologies.

## Quantitative PpIX Visualization With Optical-Sectioning Microscopy

**Figure 1:**
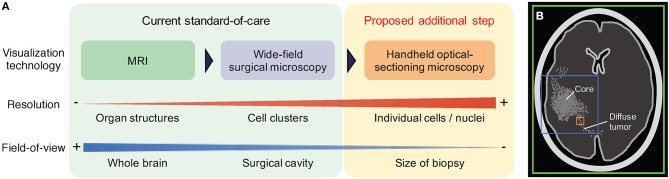
Comparison of routine and emerging imaging techniques for neurosurgical guidance. **(A)** In the current standard-of-care, pre-operative MRI is used to assess the location and size of the bulk tumor, and wide-field surgical microscopy is used intraoperatively to guide debulking. Neither method provides sufficient spatial resolution or sensitivity to effectively visualize diffuse tumors at the surgical margins. Handheld optical-sectioning microscopy is an intraoperative imaging technique that provides superior resolution and sensitivity to detect infiltrating tumor cells at the margins, and can be potentially used to quantify tumor parameters at localized regions at the final stages of resection. **(B)** The colored boxes indicate the relative field-of-view (FOV) of the imaging modalities described. Larger FOVs are advantageous to mitigate sampling errors when imaging heterogeneous tissues, but typically require trade-offs in terms of resolution and sensitivity. Green: MRI. Blue: wide-field microscopy. Orange: optical-sectioning microscopy with mosaicking.

Optical-sectioning microscopy enables cross-sectional imaging of intact tissue at shallow depths (<0.5 mm deep) by removing the background “haze” due to out-of-focus and multiply-scattered photons. A technical description of various optical-sectioning approaches has been provided in previous review articles (58–61). In general, optical-sectioning microscopy provides the superior image contrast and spatial resolution that is necessary to detect the weak and sparse PpIX fluorescence generated by LGGs and at the infiltrative margins of all diffuse gliomas (55). An increasing number of studies have showcased the feasibility to examine PpIX expression in human gliomas at the microscopic level using optical-sectioning techniques (55, 56, 62, 63). Microscopic PpIX expression in gliomas is manifested as localized subcellular foci of fluorescence, a pattern that is consistent with our current biological understanding of subcellular PpIX generation by mitochondria (43). Quantification of microscopic PpIX expression based on a tabletop line-scanned dual-axis-confocal (LS-DAC) microscopy—a high-speed, high-contrast optical-sectioning technique—has been shown to agree with conventional fluorescence histology (56), suggesting that a miniature LS-DAC device could serve as a real-time non-invasive alternative to slide-based histopathology. As detailed in a recent review (47) and summarized in Figure 1, the main trade-off for high-resolution microscopy is a limited FOV that can lead to sampling bias in glioma tissues that are often spatially heterogeneous. To mitigate this problem, handheld confocal microscopy with video-mosaicking (i.e., stitching overlapping video frames to create an extended FOV over time using image processing algorithms) have been developed to sample a tissue region comparable in size to a physical biopsy specimen (several millimeters in scale) while maintaining high resolution and contrast (57, 64–68).

## Proposed Clinical Workflow

**Figure 2:**
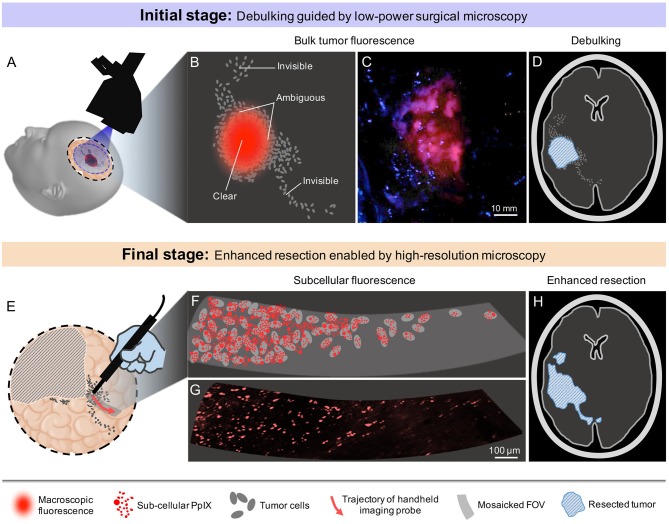
Proposed clinical workflow of 5-ALA-based fluorescence-guided neurosurgery. **(A)** Conventional low-power fluorescence surgical microscopy provides a wide FOV that often covers the entire surgical cavity. **(B)** Macroscopic PpIX fluorescence is visible at the central portions of most HGGs, but not at the infiltrative margins. **(C)** Example low-power fluorescence microscopy image of PpIX expression from a bulk HGG region. The margins of the tumor are subjectively delineated and ambiguous. **(D)** Debulking guided by macroscopic PpIX fluorescence, resulting in residual tumor burden. **(E)** High-sensitivity probe-based optical-sectioning microscopy is used to examine localized regions near the surgical margins, providing a non-invasive alternative to intraoperative frozen section histopathology. **(F)** Visualization of subcellular PpIX fluorescence can potentially enable quantification of tumor parameters in order to guide surgical decisions at the final stages of resection. **(G)** Example image of microscopic PpIX fluorescence in a HGG biopsy using high-resolution optical-sectioning microscopy. **(H)** Optimal extent of resection is achieved after iterative tumor resection guided by video-mosaicked handheld optical-sectioning microscopy.

In the current clinical workflow for 5-ALA-based FGS, glioma margins are defined by pre-operative or intraoperative magnetic resonance imaging (MRI), as well as wide-field (low-power) surgical microscopy. However, since all gliomas are diffuse and ill-defined, the contrast-enhancing regions revealed by these wide-field imaging methods (e.g., Gd-enhancement for HGGs, T2-hyperintensity for LGGs, and macroscopic PpIX fluorescence for most HGGs) are not indicative of the actual extent of tumor infiltration. While frozen-section histopathology can confirm tissue status during the course of glioma resection, this strategy is invasive (requiring a physical biopsy) and time consuming. Our hypothesis, to be investigated in future prospective studies, is that when operating on gliomas adjacent to eloquent cortical and subcortical pathways, quantitative high-resolution microscopy can be used at the final stages of resection to interrogate tumor burden and other quantitative biomarkers at multiple suspicious sites in order to optimize the EOR (including beyond the radiographic margins) without jeopardizing functional pathways. As shown in Figure 2, a specific workflow for future clinical use is provided below:

(1) At the initial stages of the surgery, standard neurosurgical methods will be used for debulking the central portions of the tumor. As the neurosurgeon approaches the radiographic or functional boundaries of the tumor (indicated by anatomical/visual cues, MRI-based neuronavigation, intraoperative stimulation mapping, and 5-ALA-based FGS using wide-field surgical microscopy), regions adjacent to non-eloquent brain can be resected more aggressively to minimize residual tumor burden.

(2) At the final stages of surgery, ambiguous regions at critical locations (e.g., near eloquent brain) will be probed with high-resolution video-mosaicked microscopy of the exposed tissue surfaces, enabling a quantitative measure of microscopic PpIX expression that should ideally correlate with clinicopathologic metrics such as tumor burden and proliferative/mitotic index in order to guide operative decision-making. This provides a non-invasive and real-time alternative to intraoperative consultation with frozen-section histology. It should be noted that similar sterile probe-based microscopy/spectroscopy strategies have been implemented during neurosurgeries, as described in several reports (69–73).

(3) Conventional surgical tools (e.g., ultrasonic aspirator, suction catheters, etc.) will be used in an iterative process with intraoperative microscopy until optimal resection has been achieved. Ideally, neuronavigation would be used to track the spatial coordinates of all surgical devices (microscope, suction catheters, etc.) to ensure good co-registration between iterative rounds of imaging and resection.

## Challenges and Future Steps

A number of translational milestones should ideally be achieved in order to bolster confidence in a high-resolution intraoperative imaging technique for adoption by surgeons. First, and perhaps the most critical step, is to establish a biological context and understanding of the pattern of sub-cellular PpIX expression that is visualized with an optical-sectioning microscope. Note that numerous studies have already shown that PpIX expression provides specific delineation of a variety of neoplasms under wide-field (low-resolution) imaging and spectroscopy (52, 74, 75), including a general correlation between PpIX concentrations and proliferative score as well as World Health Organization (WHO) histologic score (52). Preliminary studies [e.g., using high-resolution *in vivo* microscopy (55), or using fluorescent-activated cell sorting of dissociated human cells [unpublished data]] have also shown that PpIX expression at the cellular level is highly tumor-specific. However, larger-scale correlation studies are needed to improve our ability to interpret high-resolution images of PpIX in gliomas. For example, studies should ideally demonstrate a clear correlation between subcellular patterns of PpIX fluorescence and well-established clinicopathologic metrics such as tumor burden and proliferative/mitotic index (e.g., Ki-67 and pHH3 expression). Facilitated by the recent advancements in both imaging hardware [e.g., open-top light-sheet microscopy (63)] and artificial intelligence (e.g., deep-learning algorithms for classification and regression tasks), it should be possible to perform large correlative studies within a reasonable timeline. A second milestone, as mentioned previously, is to mitigate sampling bias due to tissue heterogeneity and to more-closely match the spatial precision of current surgical tools (typically several millimeters in scale). While it is technically challenging to engineer high-resolution microscopes with such large FOVs, robust computer vision algorithms have been developed and continue to be refined to stitch overlapping image frames together to create an extended FOV in real time while an imaging device is translated along the tissue surface (57, 64–68).

It bears repeating that the goal of optical-sectioning microscopy is NOT to image deeply, but rather to perform quantitative imaging near the exposed tissue surface, which requires the high contrast of an optical-sectioning device. However, in practice, the ability to image over a shallow range of depths (<150 microns) may be of practical value to identify an optimal depth where image quality and tissue integrity are maximized. Complementary imaging modalities for detecting PpIX fluorescence from deep subsurface tumors (76–78) are beyond the scope of this perspective paper. In terms of resolution, since current resection tools lack the spatial precision of a high-resolution microscope, the value of high-resolution imaging is not to enable cellular-scale resection, but to enable accurate quantification of PpIX, which in turn should correlate with relevant metrics of tumor burden/proliferation to guide surgical decisions. As with all innovative technologies, clinical validation is needed through well-powered and controlled studies. For example, “malignancy scores” based on quantitative PpIX microscopy should agree with traditional assessment methods such as histopathology and post-operative MRI, and should also be predictive of patient outcomes (e.g., recurrence). Note that for most glioma patients, adjuvant radiotherapy is a logical next-step following tumor resection. The development of technology that enables microscopic quantification of tumor burden and proliferation, and therefore identification of resection cavity regions with high-risk of tumor recurrence, could also inform postoperative radiotherapy planning and improve the efficacy of radiation-based strategies to control tumor progression.

In summary, the recent FDA approval of 5-ALA-based FGS and the advancement of fluorescence imaging technologies have provided a unique opportunity to improve glioma surgeries. We believe that handheld video-mosaicked optical-sectioning microscopy has an important role to play in improving the EOR for glioma surgeries through quantitative and reproducible delineation of the infiltrative margins of diffuse gliomas, for which current techniques fail to provide adequate guidance at the final most-critical stages of resection procedures. The successful translation of this technology will require collaborative efforts amongst multidisciplinary teams that include optical engineers, neurosurgeons, pathologists/biologists, computer scientists, industry partners, and regulatory/reimbursement stakeholders. Together with wide-field imaging techniques such as MRI and low-power surgical microscopy, we have provided a perspective that the use of intraoperative high-resolution microscopy within the surgical armamentarium should yield significant improvements in glioma patient outcomes.

## Data Availability

No datasets were generated or analyzed for this study.

## Author Contributions

All authors listed have made a substantial, direct and intellectual contribution to the work, and approved it for publication.

### Conflict of Interest Statement

The authors declare that the research was conducted in the absence of any commercial or financial relationships that could be construed as a potential conflict of interest. The handling editor declared a shared institutional affiliation, though no other collaboration, with several of the authors (YF, NS).
